# Radiographic sclerosis with intraoperative fragile bone in skeletal fluorosis: a case report

**DOI:** 10.3389/fsurg.2026.1695296

**Published:** 2026-03-31

**Authors:** Weikun Hou, Lin Liu, Wensen Jing, Chao Lu, Yangquan Hao

**Affiliations:** Department of Osteonecrosis and Joint Reconstruction, Xi’an Honghui Hospital, Health Science Center of Xi’an Jiaotong University, Xi'an, Shaan’xi, China

**Keywords:** endemic disease, osteoporosis, radiographic sclerosis, skeletal fluorosis, total knee arthroplasty

## Abstract

**Introduction:**

Skeletal fluorosis is a rare toxic osteopathy characterized by massive bone fluoride fixation. The disease is an endemic problem in some parts of the world and is the result of the prolonged ingestion or inhalation of large amounts of fluoride. The radiographic presentation is mainly characterized by bone changes, with osteocondensation and ossification of many ligaments and interosseous membranes.

**Diagnoses:**

A 59-year-old woman with skeletal fluorosis underwent staged bilateral total knee arthroplasty. Preoperative radiographs showed increased bone density, thickened and fused trabecular, a thickened cortical bone, and a narrowed marrow cavity.

**Patient concerns:**

Severe osteoporosis combined with calcification of the capsule, ligaments, and tendons was found intraoperatively, which increased both exposure difficulty and periprosthetic fracture risk.

**Lessons:**

Skeletal fluorosis is not clinically obvious and is easily misdiagnosed. It is essential to evaluate bone quality and soft tissue flexibility preoperatively in patients from endemic areas. Special exposure procedures and release techniques are useful in these cases. It is recommended that clinicians fully appreciate that skeletal fluorosis can occur in the presence of preoperative radiographic sclerosis and intraoperative osteoporosis.

## Introduction

Skeletal fluorosis is an endemic bone and joint disease caused by the consumption of high concentrations of fluoride water, food, or medicine, leading to bone and joint pain, joint stiffness, limb motor dysfunction, and skeletal deformities (1). It can be classified into the following three stages of manifestations on X-ray, according to Hagen and Grinsberg: Stage I: Bone thinning and ill-defined margins, gradually changing with increased density of bone tissue; Stage II: coarsening of the corticalis of spongious bones and periosteal appositions; Stage III: marked density, loss of trabecular structure, periostosis and osteophyte formation, and ossification and calcification of the ligaments, typically in the sacroiliacal region (2). However, the radiological manifestations do not always accurately represent the bone quality.

Here we report a case of bilateral total knee arthroplasty (TKA) with skeletal fluorosis and a paradoxical phenomenon of preoperative radiographic sclerosis and severe osteoporosis intraoperatively.

## Case report

**Figure 1:**
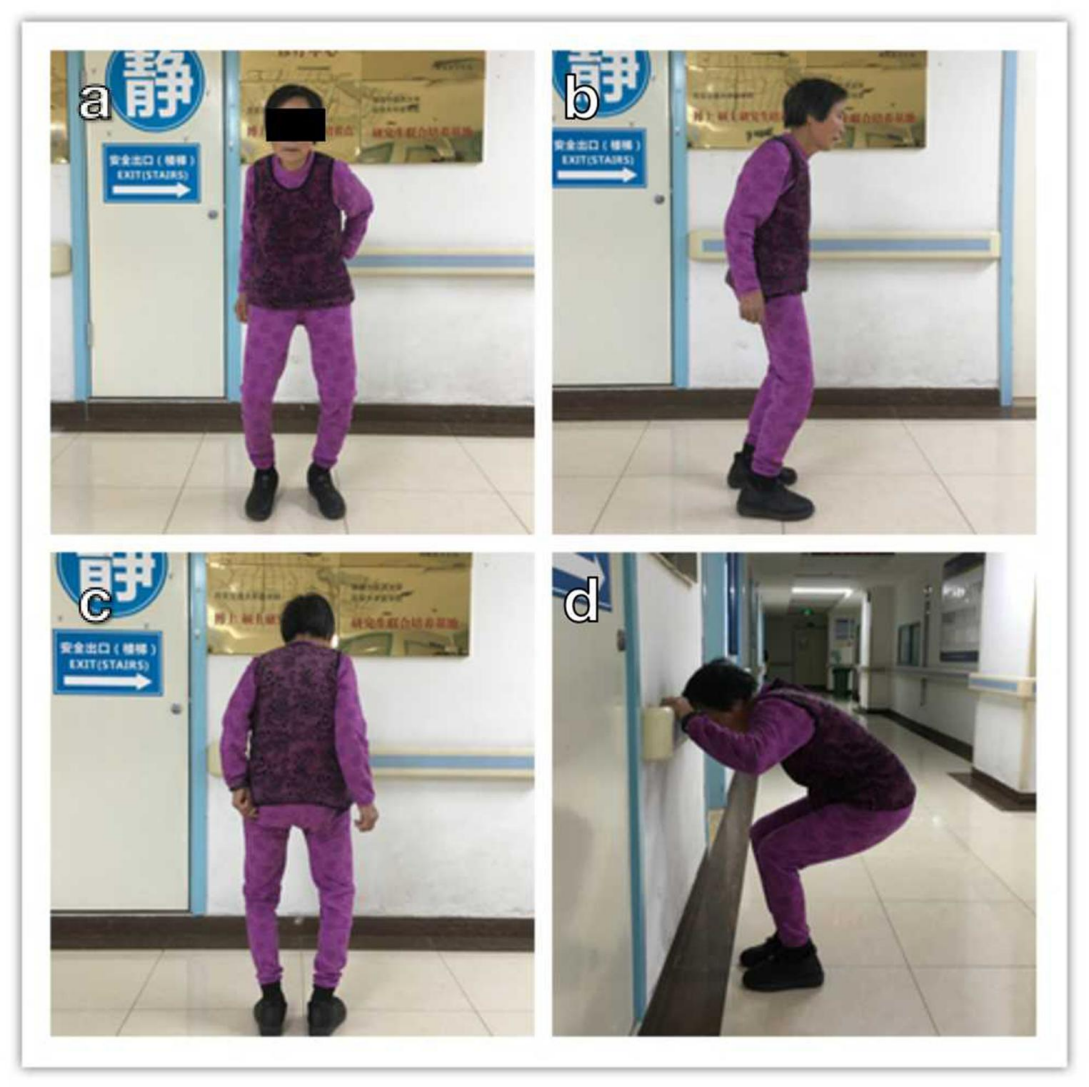
Preoperative anterior **(a)**, lateral **(b),** and posterior **(c)** views, which show bilateral knee varus deformities with flexion contracture deformities. **(d)** Lateral view of the patient with the maximum degree of knee flexion during squatting.

**Figure 2:**
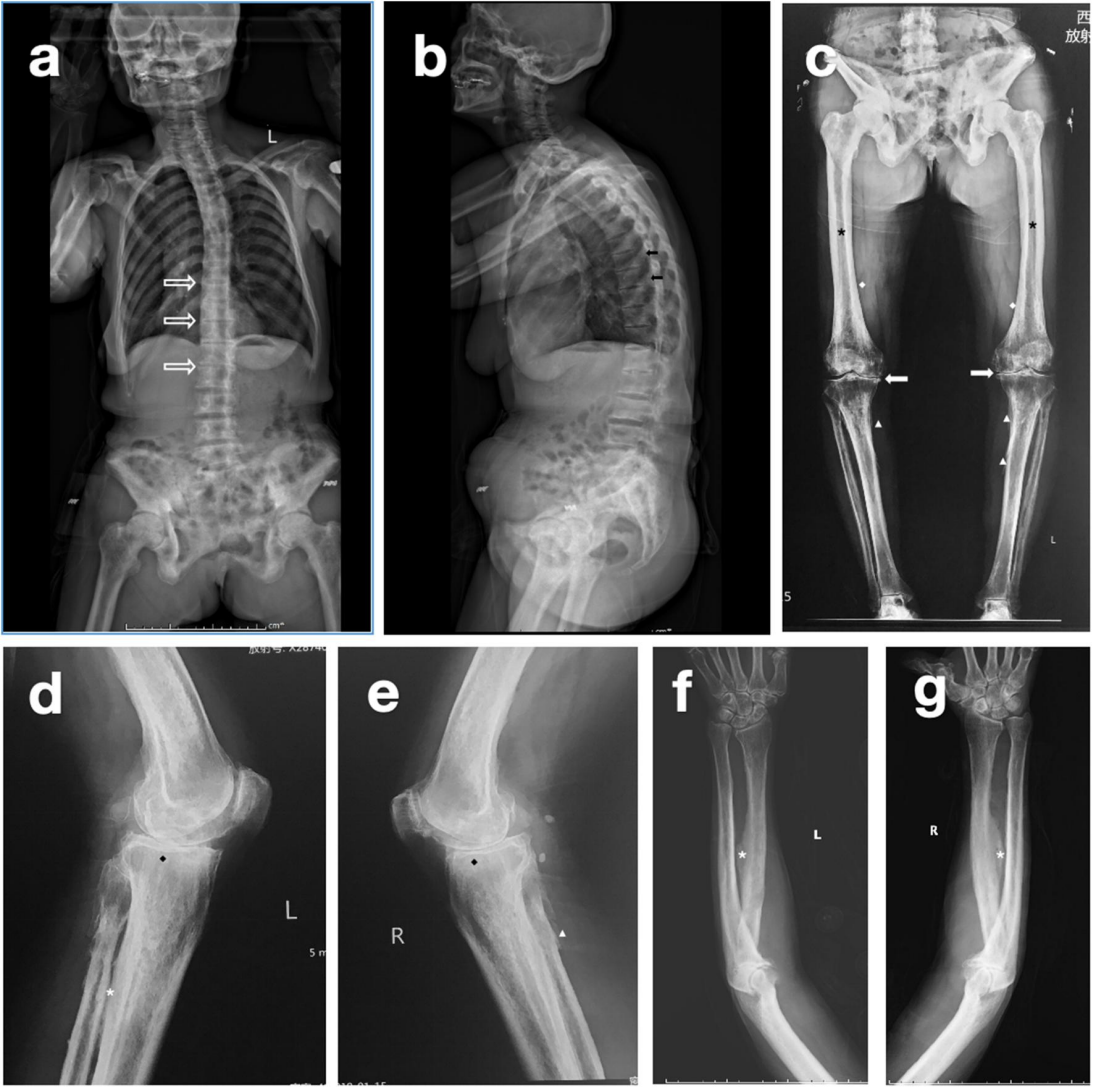
Preoperative radiographic appearance: **(a)** anteroposterior and **(b)** lateral views of the spine show typical bamboo-shaped changes (hollow white arrow) and calcification of the ligamentum flavum (black arrow) in the thoracic and upper lumbar vertebrae. **(c)** Full-length anteroposterior view of the left **(d)** and right knees. **(e)** The lateral view shows a varus deformity, a narrowed medial joint space (solid white arrow) with increased bone density, a fused trabecular bone (black rhombus), a thickened cortical bone (white rhombus), a narrowed marrow cavity (black star), ossification of ligaments (white triangle), and interosseous membranes (white star). **(f)** Anteroposterior and **(g)** lateral views of the forearm show ossification of interosseous membranes (white star).

A 59-year-old woman presented to our hospital with 15 years of knee pain, stiffness, and limited range of motion (Figure 1). She had scoliosis and typical dental fluorosis. She was born and lived in a high-fluoride area, and drank high levels of fluoride water (>4 mg/L) for several decades until recent years. Her knees had varus deformities with 30 degrees of flexion contracture. She had received oral non-steroidal anti-inflammatory drugs and intra-articular sodium hyaluronate injection treatment, but without effect. A standard preoperative evaluation was performed, and laboratory testing revealed no further abnormalities. The patient’s urinary fluoride level was 0.62 mg/L (the fluoride ion selective electrode method was used, reference range <1.6 mg/L, drinking water fluoride exposure content 0.5–1.5 mg/L; the “Standards for the Hygiene of Drinking Water” in China stipulates that the fluoride content in drinking water should not exceed 1.0 mg/L). Anteroposterior and lateral X-rays of both knee joints showed varus deformity, osteophyte formation in the tibial and femoral edges, spinous process of tibia, increased bone density, a thickened and fused trabecular bone, a thickened cortical bone, and a narrowed marrow cavity (Figures 2a–e). An X-ray of the patient’s forearm showed ossification of the interosseous membrane (Figures 2f,g). The patient was scheduled to undergo staged bilateral total knee arthroplasty, with the right knee first and an 11-month interval.

**Figure 3:**
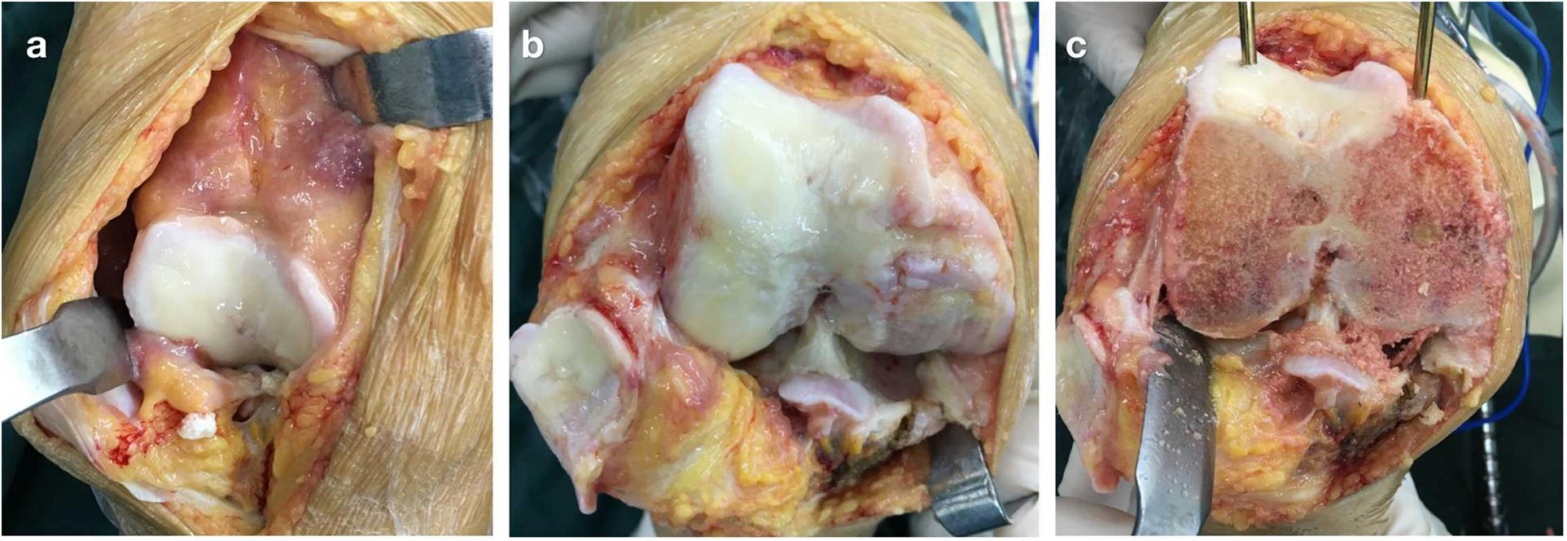
Intraoperative view of the right knee: **(a)** inflammatory synovial membrane, **(b)** cartilaginous exfoliation and subchondral sclerosis of the medial condyle of the femur and the medial plateau of the tibia, and **(c)** osteoporosis, bone cysts, and osteophyte formation were found after distal femoral resection.

**Figure 4:**
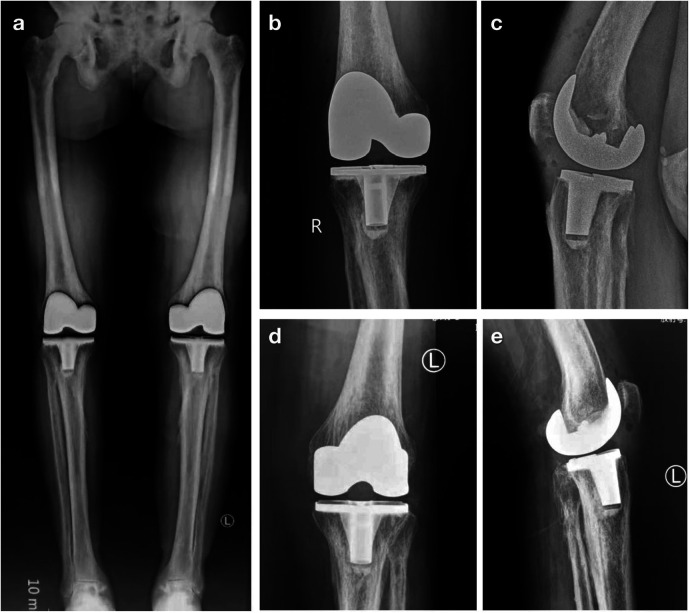
Postoperative radiographic appearance. **(a)** The full-length lower limb anteroposterior radiographic view shows restored lower limb alignment, with the right knee **(b)** anteroposterior and **(c)** lateral views and left knee **(d)** anteroposterior and **(e)** lateral views showing the excellent positioning, alignment, and angle of the prostheses.

A standard midline incision and parapatellar approach were used. Inflammatory hyperplasia of the synovial tissue and the suprapatellar fat pad were resected (Figure 3a). The deep medial collateral ligament (MCL) was elevated to allow sufficient visualization of the proximal tibia to perform a proximal tibial resection. The patella was difficult to evert in this stiff knee. The lateral patellofemoral ligament was then divided sharply, the infrapatellar fat pad was resected, the lateral patellar retinacula were released, and the patella was safely everted laterally. The medial compartment and patella were worn and the formation of osteophytes and loose bodies was observed (Figure 3b). The “femur first” technique was used. An intraoperative quality assessment of bone status was performed by a physician during bone preparation. The femur cutting block was stabilized and aligned using an intramedullary rod. The valgus correction angle was set at 7 degrees. After finishing the distal femur resection, severely osteoporotic bones (a thin cortical bone and sparse bone trabeculae of the femoral condyles, with low resistance to bone sawing) and bone cysts were found (Figure 3c). Caution was taken to avoid a fracture and tearing of ligaments. Standard tibial resection was conducted, with haptic feedback to the surgeon indicating soft bone. Thus, low drilling torque was used as the bone had low resistance to sawing, keeling, chiseling, and cementation impaction. NexGen Posterior Stabilized (PS) Total Knee Prostheses (Zimmer Biomet, Warsaw, IN, USA) were implanted (Figures 4a–e) with cement fixation. The knee capsule was closed anatomically using absorbable sutures in the deep layers and a running subcuticular closure on the skin. The wound was dressed with a sterile dressing.

**Figure 5:**
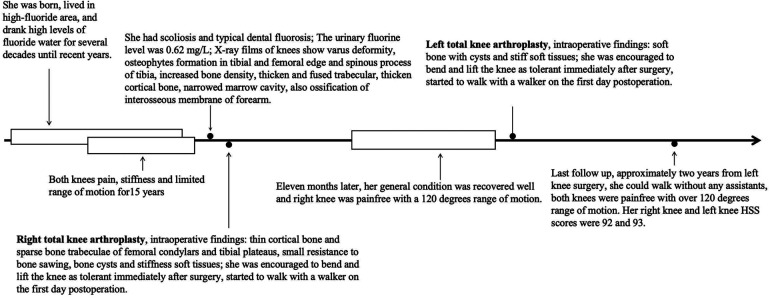
Timeline summarizing the diagnostic and surgical course.

The patient was encouraged to bend and lift the knee as tolerated immediately after surgery. She started to walk on the first day postoperation. Her right knee hospital for special surgery knee score (HSS) score increased from 54 preoperatively to 76 one week postoperatively. Eleven months later, she was ready to undergo the left knee arthroplasty. She had recovered well and her right knee was pain-free with a 120 degrees range of motion. Intraoperatively, a similar midline incision and parapatellar approach was used. After transecting the lateral patellofemoral ligament, resecting the infrapatellar fat pad, and releasing the lateral patellar retinacula, the patella was everted laterally. NexGen PS Total Knee Prostheses (Zimmer Biomet, Warsaw, IN, USA) were implanted.

Similarly, she was encouraged to bend and lift the knee as tolerated immediately after surgery and she started to walk on the first day postoperation. Postoperative radiographies showed excellent positioning, alignment, and angle of the prostheses. Her left knee HSS score increased from 58 preoperatively to 88 1 week after the left TKA. At the last follow-up, approximately 2 years after the left knee surgery, she could walk without any assistance and both knees were pain-free with ranges of motion of over 120 degrees. Her right and left knee HSS scores were 92 and 93, respectively (Figure 5). We had access to information that could identify her as an inpatient or an outpatient during follow-up. Written informed consent was obtained from the patient's son for the publication of this case report. This report was approved by the Medical Ethics Committee of Xi'an Honghui Hospital, Health Science Center of Xi'an Jiaotong University (No. 201902059).

## Discussion

Fluorine is a common chemical element and the lightest member of the halogen elements, with fluoride its ionic form (3). The concentration of fluoride in the body is strongly associated with diet, occupational exposure, environmental conditions, medication, and the health status of the population (4). It is assumed that approximately 93%–97% of fluoride in the body is accumulated in hard tissues, such as bones and teeth (5). Fluoride, which is widely added to toothpaste and the water supply, protects teeth from decay through demineralization and remineralization (6).

Sodium fluoride affects bone strength in different ways. First, approximately 50% of fluoride is incorporated into hydroxyapatite crystals, replacing the hydroxide ion and altering the size and structure of the crystals. Second, fluoride may influence bone turnover by regulating certain factors, such as Runx2 and receptor activator of nuclear factor kappa-B ligand (7). It also affects the expression of osteocalcin (OCN) and osteoprotegerin and may cause an increase in osteoblast activity (8).

The functions of sodium fluoride in bone are biphasic. Different concentrations of fluoride can regulate bone glycoprotein metabolism and mineralization differentially (9). A low concentration of fluoride can have a therapeutic role in osteoporosis, increasing the proliferation of osteoblasts; stimulating the expression of fibronectin, vimentin, Runx2, and osterix (OSX) to promote the expression of OCN and osteopontin (OPN) for osteogenetic process; and inhibiting the function of osteoclasts. Prolonged exposure to large amounts of fluorine results in the deposition of fluoride in the bones. However, exposure to a high concentration of fluoride over the years inhibits Runx2 and induces oxidative stress and DNA damage in osteoblasts, resulting in skeletal fluorosis. A high bone mass density does not necessarily indicate strong bones, as, although fluoride stimulates bone formation, the bone that is formed is of a lower quality and can break more easily (10).

Skeletal fluorosis is an endemic disease in areas where the concentrations of fluorine are higher due to increased activity of flowing gas and rock and is caused by an excessive accumulation of fluoride and characterized by bone outgrowths with osteocondensation and later ossification of many ligaments and interosseous membranes (11). Its symptoms are chronic diffuse skeletal pain and joint stiffness, which mimic the symptoms of osteoarthritis (12). Skeletal fluorosis can produce bone changes with joint space narrowing and osteophyte formation that resemble osteoarthritis, making it difficult to diagnose in the absence of detectable osteosclerosis in the spine (13, 14). Skeletal fluorosis is still a serious concern due to it being underdiagnosed and its expensive treatment (15).

TKA is the gold-standard method to treat end-stage knee disease, such as osteoarthritis, rheumatoid arthritis, spontaneous osteonecrosis of the knee, ankylosing spondylitis, and Kaschin-Beck disease; however, to our knowledge, few articles have described TKA in patients with skeletal fluorosis (16, 17). A proper diagnosis of skeletal fluorosis is critical for preoperative planning in total knee arthroplasty. The increased density noted on X-rays often appears to be due not to a true increase in bone mineralization, but rather to exostotic thickening of the bone (18). The paradox of high radiographic sclerosis in preoperative images and an intraoperative finding of severe osteoporosis may mislead the surgeon and cause unexpected complications, such as a periprosthetic fracture or tearing of ligaments (19). Paiste et al. reported one case of intraoperative femoral shaft fracture, which was caused by a drill breaching the medial femoral cortex when opening the femoral canal (16).

Skeletal fluorosis causes calcification of the capsule, ligaments, tendons, fat pad, and synovium, reducing their elasticity (20). It is difficult to expose the joint and evert the patella using the routine maneuver. In this case, we first extended the skin incision distally and proximally. We then transected the lateral patellofemoral ligament, resected the infrapatellar fat pad, and released the lateral patellar retinacula before everting the patella. There is another concern that the periarticular contracture may continue and lead to a flexion contracture deformity postoperatively. It is important to acquire full extension or 3–5 degrees of over-extension during the surgery, either by increasing the distal femoral resection or releasing the posterior capsules. The postoperative rehabilitation process should be personalized due to the stiff soft tissues and fragile bones. In this case, the patient was encouraged to bend and extend her knees as tolerated immediately after the surgeries and maintain daily exercise to maintain full extension and increase their range of motion.

## Limitations of the study

First, the sample size is too small. This is a rare case, as the patient lived in a remote and high-fluoride area and drank high levels of fluoride water for several decades until recently, when her drinking water was replaced by tap water. Despite screening all the cases that received arthroplasty in our hospital, no similar cases have been found. Second, no serum or bone metabolism indices were included and bone mineral density was not evaluated.

## Conclusion

Skeletal fluorosis can present paradoxically with high radiographic sclerosis and osteoporosis intraoperatively, which may mislead the surgeon and cause unexpected complications during total knee arthroplasty. Proper diagnosis and meticulous surgical soft tissue release and bone resection are critical to reduce complications and improve clinical function.

## Data Availability

The raw data supporting the conclusions of this article will be made available by the authors, without undue reservation.
